# *Let-7* Sensitizes *KRAS* Mutant Tumor Cells to Chemotherapy

**DOI:** 10.1371/journal.pone.0126653

**Published:** 2015-05-06

**Authors:** Xin Dai, Ying Jiang, Chalet Tan

**Affiliations:** Department of Pharmaceutical Sciences, Mercer University, Atlanta, Georgia, United States of America; University of Nebraska Medical Center, UNITED STATES

## Abstract

*KRAS* is the most commonly mutated oncogene in human cancers and is associated with poor prognosis and drug resistance. *Let-7* is a family of tumor suppressor microRNAs that are frequently suppressed in solid tumors, where *KRAS* mutations are highly prevalent. In this study, we investigated the potential use of *let-7* as a chemosensitizer. We found that *let-7b* repletion selectively sensitized *KRAS* mutant tumor cells to the cytotoxicity of paclitaxel and gemcitabine. Transfection of *let-7b* mimic downregulated the expression of mutant but not wild-type KRAS. Combination of *let-7b* mimic with paclitaxel or gemcitabine diminished MEK/ERK and PI3K/AKT signaling concurrently, triggered the onset of apoptosis, and reverted the epithelial-mesenchymal transition in *KRAS* mutant tumor cells. In addition, *let-7b* repletion downregulated the expression of β-tubulin III and ribonucleotide reductase subunit M2, two proteins known to mediate tumor resistance to paclitaxel and gemcitabine, respectively. *Let-7* may represent a new class of chemosensitizer for the treatment of *KRAS* mutant tumors.

## Introduction

*KRAS* mutations occur in approximately 20% of all human cancers and are particularly prevalent in pancreatic ductal adenocarcinoma (PDAC, ~90%), non-small cell lung cancer (NSCLC, ~25%) and colorectal cancer (~40%) [1]. As a driver oncogene, constitutively activated *KRAS* transduces cascades of parallel phosphorylation reactions in RAF/MEK/ERK and PI3K/AKT/mTOR pathways among others, culminating with uncontrolled cell proliferation, evasion of apoptosis, and metastasis. Consequently, intensive effort has been made to target mutant *RAS* for the treatment of cancer. A number of strategies have been pursued in order to thwart *KRAS*-driven oncogenesis, including the combination of kinase inhibitors (such as PI3K and MEK inhibitors) to simultaneously repress downstream RAS effectors [2,3], the inhibitors that directly attenuate the kinase activity of mutant KRAS [4,5], and the small interference RNAs (siRNAs) that specifically knockdown mutant *KRAS* [6]. To date, none of these approaches has been approved for clinical uses. Treatment of *KRAS* mutant tumors still largely relies on conventional chemotherapy, which commonly results in poor response rate and development of drug resistance in cancer patients.

MicroRNAs (miRNAs) are endogenous, single-stranded noncoding RNAs (~22 nucleotides in length) that control gene expression at the post-transcriptional level [7]. By imperfectly base-pairing with the 3’-untranslated region (3’-UTR) of the target messenger RNAs (mRNAs), miRNAs suppress protein translation by either impeding the translation initiation or accelerating the degradation of mRNAs. It is estimated that 60% of all human protein-coding genes are the direct targets of miRNAs [8]. Since the first discovery over two decades ago, more than 1,000 human miRNAs have been identified, many of which are aberrantly expressed in tumor cells and play causative roles in tumorigenesis and tumor progression.

Consisted of 13 related miRNAs located on 9 different chromosomes, the human *let-7* microRNA family is crucially involved in cell differentiation and proliferation during development by directly targeting a large number of oncogenes including *RAS*, *HMGA2* and *MYC* [9]. *Let-7* family members are tumor suppressor miRNAs abundantly expressed in differentiated normal tissues, but are frequently lost in human cancers including NSCLC and PDAC [10,11]. Notably, the expression of *let-7a* and *let-7f* is inversely correlated with the survival of NSCLC patients [11]. In NSCLC cells, transfection of *let-7a*, *let-7b*, *let-7c*, *let-7d* and *let-7g* all potently reduced KRAS expression and impaired cell proliferation, reflecting the similar biological functions of the *let-7* family members [12]. Ectopic expression of *let-7b* and *let-7g* has been shown to repress tumor initiation in *KRAS* mutant NSCLC in mouse models [13,14]. However, *let-7* restoration does not trigger apoptosis in *KRAS* mutant tumor cells, limiting its anticancer potency against pre-established tumors [15,16].

In the current work, we explored the therapeutic potential of *let-7* as a chemosensitizer for conventional chemotherapy. Our study reveals that *let-7b* repletion selectively downregulates mutant KRAS expression and potentiates the anticancer activity of paclitaxel and gemcitabine in *KRAS* mutant tumor cells, which is accompanied by attenuated cell proliferation, enhanced apoptosis and the reversal of the epithelial-mesenchymal transition (EMT) phenotype in tumor cells.

## Materials and Methods

### Cell Culture

Human NSCLC cell lines A549 and NIH-H1975, human PDAC cell lines Panc-1 and BxPC-3, human breast cancer cell lines MDA-MB-231 and MCF-7, human normal lung fibroblast cell line MRC-5 and human normal breast epithelial cell line MCF-10A were purchased from American Type Culture Collection (ATCC, Manassas, VA). A549 cells were cultured in F-12K (Life Technologies, Grand Island, NY). NIH-H1975 and BxPC-3 cells were cultured in RPMI 1640 (Life Technologies). Panc-1, MRC-5, MDA-MB-231 and MCF-7 cells were cultured in DMEM (Life Technologies). All above medium was supplemented with 10% fetal bovine serum (FBS, Life Technologies), 1% penicillin and streptomycin (Life Technologies). MCF-10A cells were cultured in DMEM/F12 (Life Technologies) supplemented with 5% fetal bovine serum, 20 ng/mL epidermal growth factor (EGF) (R&D Systems, Minneapolis, MN), 10 μg/mL insulin (Life Technologies), 0.5 mg/mL hydrocortisone (Sigma, St. Louis, MI), 100 ng/mL cholera toxin (Sigma) and 1% penicillin and streptomycin. All cells were cultured at 37°C in 5% CO_2_ incubator.

### Cell transfection and treatments

*Let-7b-5p* mimic and the non-targeting (scramble oligonucleotides) control were purchased from Bioneer (Alameda, CA). Transfection was carried out using Lipofectamine 2000 (Life Technologies) according to the manufacturer’s procedures. In brief, cells were 60–80% confluent at the time of transfection. *Let-7b* mimic or the scramble control was mixed gently with Lipofectamine 2000 diluted in the Opti-MEM reduced serum medium (Life Technologies), and incubated at room temperature for 25 minutes before treating the cells at a final concentration of 50 nM. The transfected cells were incubated for 6 hours, the medium was then replaced with fresh growth medium supplemented with 2% serum but no antibiotics. Twenty four hours post transfection, the cells were treated with varying concentrations of paclitaxel or gemcitabine for 48 hours.

### Cell viability assay and analysis of combination effects

Cells were seeded at a density of 5,000–8,000 cells/well in 96-well plates, and were treated in triplicates with varying concentrations of paclitaxel (0.1–50 nM) or gemcitabine (5–150 nM or 5–150 μM) individually, or in combination with *let-7b* mimic (50 nM) or the scramble control (50 nM) as described above. Cells were then fixed with 1% glutaraldehyde, stained with 0.1% crystal violet, and dissolved in 10% acetic acid. The absorbance was quantified at 595 nm on a plate reader. The relative cell viability was calculated as the percentage of the absorbance of the treated *vs*. the untreated wells. IC_50_ of paclitaxel or gemcitabine was calculated by GraphPad Prism 5 (GraphPad Software, La Jolla, CA).

The combination index (CI) between *let-7b* and paclitaxel or gemcitabine was calculated by the CompuSyn software (ComboSyn, Paramus, NJ) [17]. The CI value was then divided into three categories: CI < 0.9, CI = 0.9–1.1, or CI > 1.1, which indicated synergistic, additive, and antagonistic effects, respectively [18].

### RNA extraction, cDNA synthesis, and quantitative real-time PCR (qRT-PCR)

Cells were transfected with *let-7b* mimic or the scramble control (50 nM) for 24 hours. The cells were then incubated with paclitaxel (20 nM) or gemcitabine (50 nM in NIH-H1975, A549 and BxPC-3 cells, 40 μM in Panc-1 cells) for 48 hours. Total RNA of cells was extracted using TRIzol (Life Technologies). The PrimeScript RT Reagent Kit (Clontech, Mountain View, CA) was used for the reverse transcription of cDNA according to the manufacturer's procedures. The reactions were incubated first at 16°C for 30 minutes, at 42°C for 30 minutes, and then inactivated by incubation at 85°C for 5 minutes.

The expression of *let-7b* was examined by qRT-PCR using TaqMan miRNA probes (Applied Biosystems, Foster City, CA) and Premix Ex Taq kit (Clontech) according to the manufacturer’s procedures. In these experiments, the relative expression level of *let-7b* in the cells was normalized to that of *U6* snRNA, a ubiquitously expressed small nuclear RNA. The expression of mRNAs was examined by qRT-PCR using the forward and reverse primers (Eurofins, Ebersberg, Germany) and EvaGreen dye (Biotium, Hayward, CA) according to the manufacturer’s protocol, and was normalized by that of β-actin in each sample. Both EvaGreen and TaqMan qPCRs were conducted using the ABI 7500 RT-PCR system (Applied Biosystems) and all of the reactions were run in triplicate. The reactions were incubated at 95°C for 5 minutes, followed by 40 cycles of 95°C for 15 seconds and 60°C for 1 minute. A comparative threshold cycle (ΔCT) method was used to compare each treatment with the internal control, and the values are expressed as 2^-ΔΔCT^. The sequences of the primers are listed in S1 Table.

### Apoptosis assay

Apoptosis was assessed by detecting the externalization of phosphatidyl serine using Annexin V (BD Biosciences, San Jose, CA). Following miRNA transfection and drug treatment as described above, A549 or Panc-1 cells were harvested and stained with Annexin V-FITC and propidium iodide (PI). After incubation for 15 minutes at room temperature, the samples were analyzed using an Accuri C6 Flow Cytometer System (BD Biosciences).

### Cell cycle analysis

Following miRNA transfection and drug treatment as described above, A549 or Panc-1 cells were harvested. The treated cells (1 × 10^6^) were fixed in 70% ethanol at 4°C overnight, washed with cold PBS, resuspended in a staining solution (0.1% Triton-X-100, 50 μg/ml PI and 1 mg/ml RNase A) for 30 minutes at room temperature, and analyzed by fluorescence cytometry using a BD Accuri C6 Flow Cytometer System. The DNA content distribution was analyzed using the FlowJo 9.3.1 software (Tree Star, Ashland, OR).

### Colony formation assay

Following miRNA transfection and drug treatment as described above, A549 or Panc-1 cells were harvested and re-seeded in triplicate in 12-well plates at a density of 100 cells/well. The plates were incubated at 37°C for 14 days. The adherent cell colonies were fixed with 1% glutaraldehyde and stained with 0.1% crystal violet at room temperature for 30 minutes.

### Scratch wound healing assay

Following miRNA transfection and drug treatment as described above, A549 or Panc-1 cells were harvested and re-seeded to grow to full confluence in 6-well culture plates. The cell monolayers were scratched with 200-μL pipette tips. The wounded monolayers were cultured in 5% FBS media for 24 hours. Closure of the wounded areas was observed under an inverted microscope (Olympus, Tokyo, Japan) at 0, 12 and 24 hours, and quantified using ImageJ software (NIH, Bethesda, MD).

### Invasion Assay

Cell invasion was evaluated using a Boyden chamber system with a polycarbonate membrane (8-μm pore size; Corning, New York, NY). For the invasion assay, the chamber inserts were pre-coated with 250 μg/mL matrigel (BD Biosciences). Following miRNA transfection and drug treatment as described above, A549 or Panc-1 cells (5×10^4^) were suspended in serum-free DMEM and seeded in the chamber inserts, and the lower chamber was filled with DMEM supplemented with 10% FBS. The cells were incubated for 40 hours. The invading cells on the bottom of the chamber inserts were fixed with 1% glutaraldehyde and stained with 0.1% crystal violet, and photographed under an inverted microscope.

### Western blot analysis

Proteins from the whole cell lysates were resolved by 10–15% SDS-PAGE and transferred onto polyvinylidene difluoride membranes (Bio-Rad Laboratories, Hercules, CA). The membranes were probed at 4°C overnight with antibodies for KRAS, ribonucleotide reductase subunit M2 (RRM2), Snail 1 (Santa Cruz, Dallas, TX), phospho-AKT, AKT, PARP, phospho-ERK-1/2, ERK 1/2, phospho-MEK, MEK, cleaved caspase-3, caspase-3, BCL-2, E-cadherin, vimentin, HMGA2 (Cell Signaling, Danvers, MA) and β-tubulin III (TUBB3, Abcam, Cambridge, MA). Densitometry of the protein bands was quantified using ImageJ software, which was normalized by β-actin level (Santa Cruz).

### Statistical analysis

Data were presented as the mean ± SEM from at least 3 independent experiments. The Student’s t-test was used to compare the statistical difference between the two tested groups. *P* value of less than 0.05 was considered statistically significant.

## Results

### *Let-7b* restoration selectively enhances the chemosensitivity of *KRAS* mutant tumor cells

**Fig 1:**
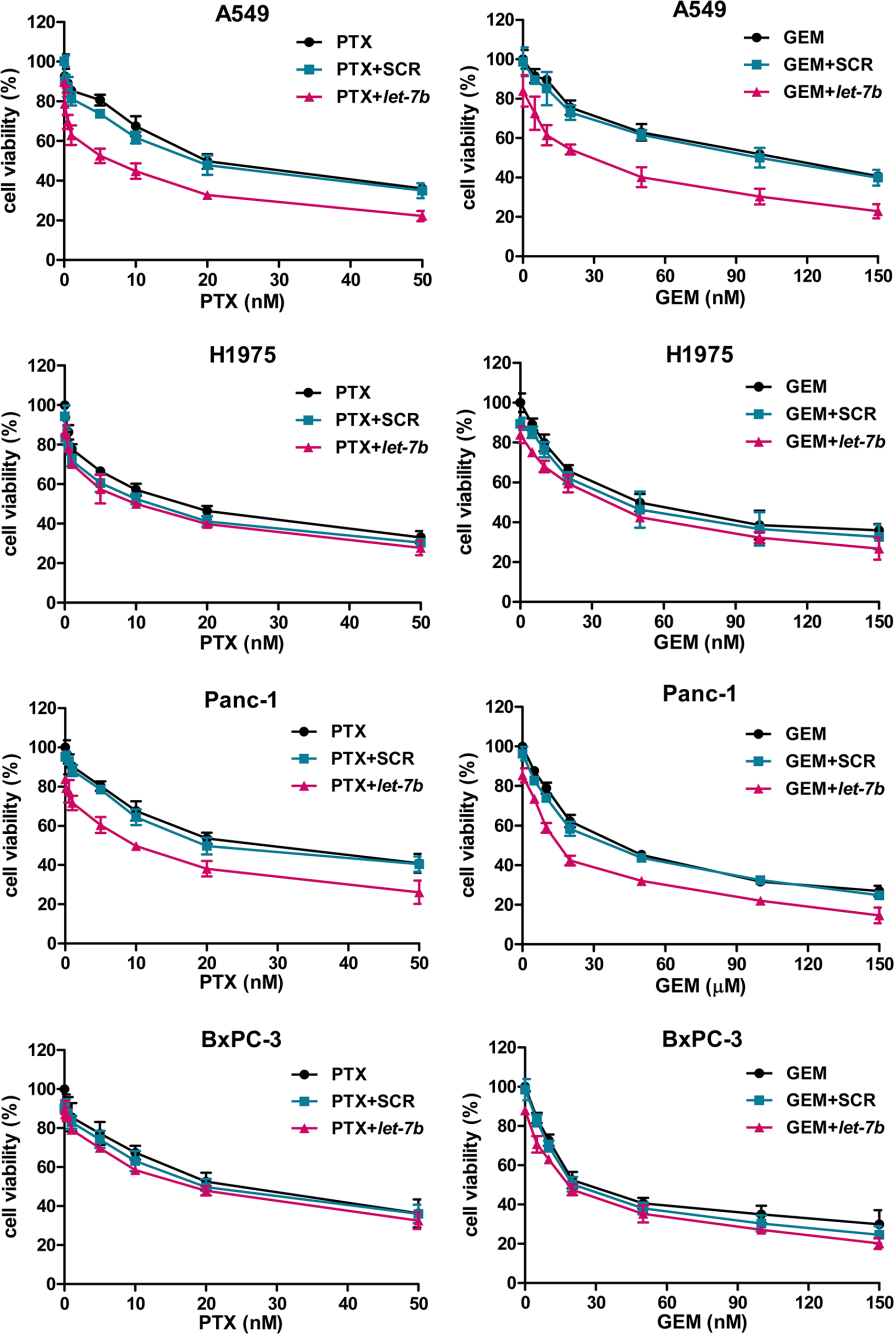
*Let-7b* repletion selectively chemosensitizes *KRAS* mutant tumor cells. NSCLC cells A549 (*KRAS* mutant) and NIH-H1975 (*KRAS* wild-type), PDAC cells Panc-1 (*KRAS* mutant) and BxPC-3 (*KRAS* wild-type) were transfected with *let-7b* mimic (50 nM) or the scramble control (SCR, 50 nM) for 6 hours. The cells were then treated with paclitaxel (PTX, 0.1–50 nM) or gemcitabine (GEM, 5–150 nM or 5–150 μM) for 48 hours. Cell viability was assessed by the crystal violet assay. Each experiment was carried out at least 3 times. Data represent the mean ±SEM.

To explore the therapeutic potential of *let-7* as a chemosensitizer, we assessed the effect of *let-7b* repletion on the cytotoxicity of paclitaxel and gemcitabine in NSCLC cells (A549 and NIH-H1975), PDAC cells (Panc-1 and BxPC-3) and breast cancer cells (MDA-MB-231 and MCF-7). The effect in the normal lung fibroblast cell line MRC-5 and the normal breast epithelial cell line MCF-10A were also evaluated. We chose to study paclitaxel and gemcitabine because of their frequent use as monotherapies and in combination therapies for the treatment of solid tumors. We found that transfection of *let-7b* mimic pronouncedly chemosensitized A549, Panc-1 and MDA-MB-231 cells, all of which harbor *KRAS* mutations, but only slightly affected *KRAS* wild-type NIH-H1975, BxPC-3, MCR-5, MCF-7 and MCF-10A cells (Fig 1 and S1 Fig). Transfection of *let-7b* mimic decreased the IC_50_ of paclitaxel from 21 nM to 4 nM in A549 cells, from 25 nM to 8 nM in Panc-1 cells, and from 10 nM to 4 nM in MDA-MB-231 cells, while the reduction was less than 30% in *KRAS* wild-type cells (Table 1 and S2 Table). Likewise, co-treatment with *let-7b* mimic reduced the IC_50_ of gemcitabine to a much greater extent in *KRAS* mutant tumor cells than in *KRAS* wild-type cells. As a single agent, *let-7b* mimic caused less than 15% inhibition in cell proliferation irrespective of *KRAS* status (Fig 1 and S1 Fig). As a negative control, the scramble control minimally influenced the cytotoxicity of paclitaxel or gemcitabine in all cell lines.

**Table 1:** Effect of *let-7b* restoration on the cytotoxicity of paclitaxel and gemcitabine in *KRAS* mutant (MT) and wide-type (WT) cell lines.

	IC_50_ of Paclitaxel			IC_50_ of Gemcitabine		
	+ scramble	+ *let-7b*	CI	+ scramble	+ *let-7b*	CI
**A549 (*KRAS* MT)**	21 ± 1 nM	4 ±1 nM	0.46	92 ± 2 nM	25 ± 1 nM	0.57
**H1975 (*KRAS* WT)**	11 ± 1 nM	9 ±1 nM	1.04	48 ± 1 nM	37 ± 1 nM	0.98
**Panc-1 (*KRAS* MT)**	25 ± 1 nM	8 ± 1 nM	0.36	36 ± 1 μM	17 ± 1 μM	0.47
**BxPC-3 (*KRAS* WT)**	22 ± 1 nM	17 ±1 nM	1.01	30 ± 1 nM	22 ± 1 nM	0.96

CI: combination index; CI < 0.9 indicates synergism; CI = 0.9–1.1 indicates additivity, and CI > 1.1 indicates antagonism.

To determine whether *let-7b*/paclitaxel or *let-7b*/gemcitabine combination resulted in synergistic effect, the combination index was calculated from the proliferation data generated in each cell line. This analysis showed that the combination indices of *let-7b*/paclitaxel and *let-7b*/gemcitabine scored well below 1.0 in *KRAS* mutant A549, Panc-1 and MDA-MB-231 cells, signifying a synergistic response (Table 1 and S2 Table). By contrast, in *KRAS* wild-type NIH-H1975, BxPC-3, MCR-5, MCF-7 and MCF-10A cells, the combination indices were around 1.0, pointing to an additive response. Together, these results indicate that *let-7b* repletion selectively sensitizes *KRAS* mutant cells to the cytotoxicity of paclitaxel and gemcitabine.

### *Let-7b* selectively downregulates mutant KRAS expression

**Fig 2:**
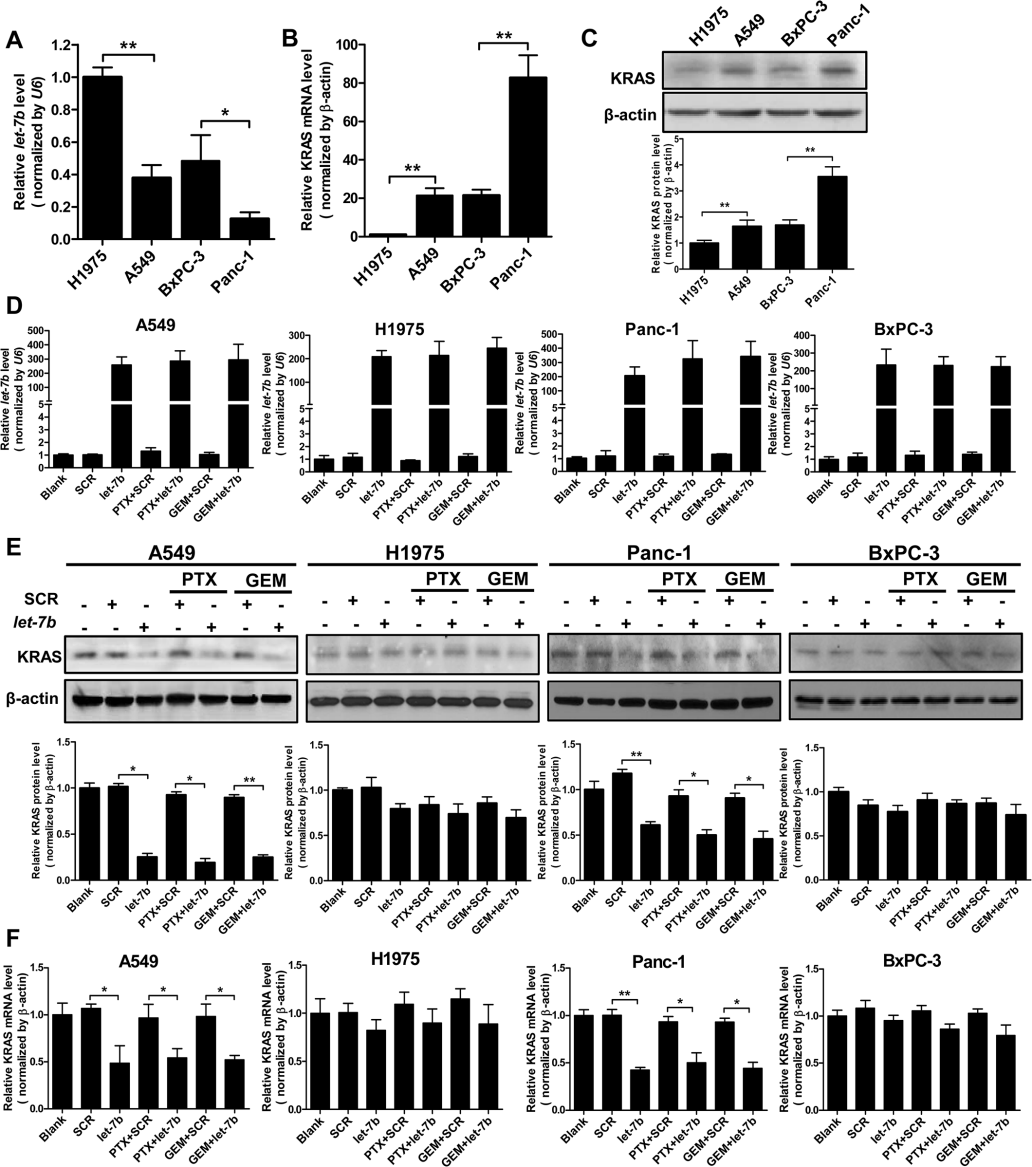
*Let-7b* selectively downregulates mutant KRAS expression. The endogenous levels of *let-7b* (A), KRAS mRNA (B) and KRAS protein (C) in NIH-H1975, A549, BxPC-3 and Panc-1 cells. The expression of *let-7b* (D), KRAS protein (E) and KRAS mRNA (F) in the cells transfected with *let-7b* mimic alone or in combination with PTX or GEM. Each experiment was carried out at least 3 times. Data represent the mean ±SEM. *, *p* < 0.05; **, *p* < 0.01.

To understand the differential effect of *let-7b* on the wild-type *vs*. mutant *KRAS* tumor cells in response to paclitaxel and gemcitabine, we first compared the endogenous levels of *let-7b* and KRAS in these cell lines. As shown in Fig 2A, the expression of *let-7b* was highest in NIH-H1975 and lowest in Panc-1 cells. Inversely, KRAS mRNA and protein levels were lowest in NIH-H1975 and highest in Panc-1 cells (Fig 2B and 2C). A549 and BxPC-3 cells expressed comparable levels of endogenous *let-7b* and KRAS. In each tissue type, there was an overall trend of lower *let-7b* and higher KRAS expression associated with mutant *KRAS*, which is consistent with the literature [19,20]. Transfection of *let-7b* mimic restored intracellular *let-7b* to a similar level in all four cell lines regardless of *KRAS* mutational status or drug exposure (Fig 2D).

Next, we examined KRAS expression in response to *let-7b*/cytotoxin treatment. We found that irrespective of paclitaxel or gemcitabine exposure, transfection of *let-7b* mimic markedly reduced KRAS expression at both the protein and mRNA levels in *KRAS* mutant A549 and Panc-1 cells (Fig 2E and 2F), whereas the effect was insignificant in *KRAS* wild-type NIH-H1975 and BxPC-3 cells. These results strongly suggest that mutant *KRAS* is more susceptible to the negative modulation by *let-7b* than the wild-type *KRAS*.

### Combination of *let-7b* with cytotoxins potentiates the blockage in MEK/ERK and PI3K/AKT signaling and promotes apoptosis in *KRAS* mutant tumor cells

**Fig 3:**
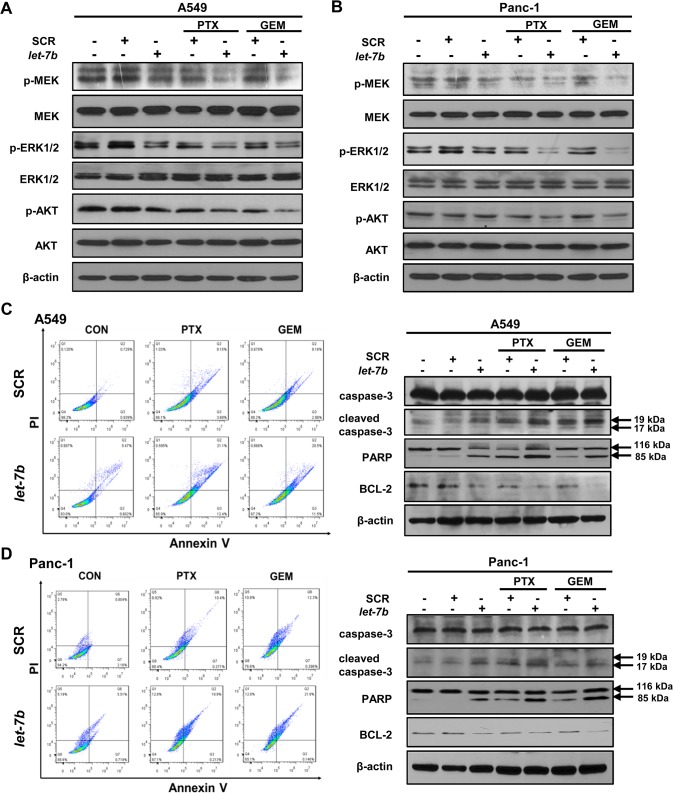
*Let-7b*/cytotoxin combination blocks mutant KRAS signaling and promotes apoptosis. The effect of *let-7b* mimic, paclitaxel or gemcitabine on the phosphorylation of MEK, ERK1/2, and AKT in *KRAS* mutant A549 (A) and Panc-1 cells (B). The apoptotic cells were detected by flow cytometry using Annexin V-FITC and PI dual staining, and the apoptotic protein markers caspase-3, PARP and BCL-2 were assessed by Western blotting in A549 (C) and Panc-1 cells (D). Each experiment was carried out at least 3 times.

Mutant KRAS constitutively activates MEK/ERK and PI3K/AKT signaling pathways, both of which are pivotal to the survival and proliferation of tumor cells [21,22]. To functionally validate the downregulation of mutant KRAS observed above, the effect of *let-7b*/cytotoxin treatment on MEK/ERK and AKT signaling was investigated. Consistent with the decreased mutant KRAS protein in A549 and Panc-1 cells (Fig 2E), transfection of *let-7b* mimic reduced the phosphorylation of MEK and ERK1/2, two RAS downstream effectors (Fig 3A). Importantly, combination of *let-7b* mimic and paclitaxel or gemcitabine diminished the phosphorylation of MEK, ERK1/2 and AKT to a much greater extent than *let-7b* mimic or either drug individually (Fig 3A and 3B). By contrast, *let-7b* mimic, paclitaxel or gemcitabine either alone or in combination failed to appreciably inhibit MEK/ERK or PI3K/AKT signaling in *KRAS* wild-type NIH-H1975 and BxPC-3 cells (S2 Fig).

Furthermore, two apoptotic markers, the cleaved caspase-3 and PARP, were more strongly induced in the cells co-treated with *let-7b*/paclitaxel or *let-7b*/gemcitabine than either agent alone, which was accompanied by the reduced expression of BCL-2, an anti-apoptotic protein (Fig 3C and 3D). In line with the Western blotting data, the results from Annexin V/PI-staining showed that combination of *let-7b* mimic with paclitaxel or gemcitabine nearly doubled the apoptotic cell population (~20%) in both A549 and Panc-1 cells, compared to those treated with either drug alone (Fig 3C and 3D). As a single agent, *let-7b* mimic induced a minor surge in apoptotic cells (~5%), in agreement with its modest cytotoxicity observed in cell proliferation assay (Fig 1). Collectively, these data indicate that combination of *let-7b* mimic with paclitaxel or gemcitabine potently blocks MEK/ERK and PI3K/AKT signaling, leading to increased apoptosis in *KRAS* mutant cells.

### *Let-7b* cooperates with cytotoxins to block cell cycle progression and inhibit colony formation of *KRAS* mutant tumor cells

**Fig 4:**
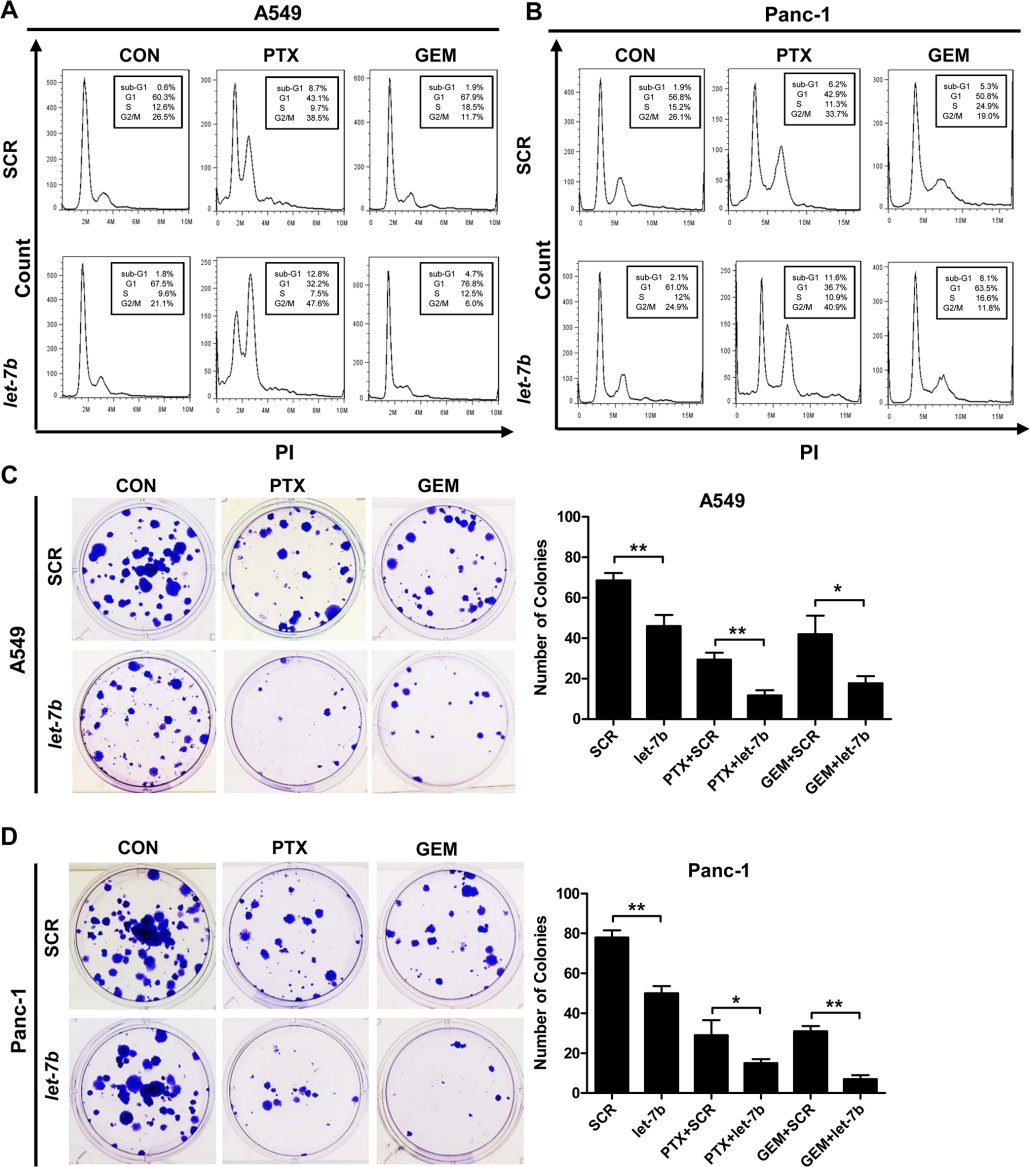
*Let-7b*/cytotoxin combination blocks cell cycle progression and inhibits colony formation of *KRAS* mutant tumor cells. The effect of *let-7b* mimic, paclitaxel or gemcitabine on cell cycle progression in A549 (A) and Panc-1 cells (B) was evaluated by flow cytometry. The effect of *let-7b* mimic, paclitaxel or gemcitabine on colony formation of A549 (C) and Panc-1 cells (D) was visualized by crystal violet staining. Each experiment was carried out at least 3 times. Data represent the mean ± SEM. *, *p* < 0.05; **, *p* < 0.01.

The *let-7* family is reported to impact on cell cycle progression and proliferation through negatively regulating multiple oncogenes [23]. To further assess the concerted effect of *let-7b* and cytotoxins on cell proliferation, we performed DNA content analysis by flow cytometry. As shown in Fig 4A and 4B, transfection of *let-7b* mimic in A549 and Panc-1 cells caused an accumulation in G_1_ phase and a corresponding reduction in S and G_2_/M phases, which is consistent with the role of *let-7* as a negative regulator of G_1_-to-S phase transition. When combined with paclitaxel, a microtubule inhibitor that caused potent G_2_/M phase arrest, the treatment elicited even more severe G_2_/M phase arrest. On the other hand, combination of *let-7b* mimic with gemcitabine, a nucleoside analog that caused S phase accumulation, arrested the cell cycle at G_1_ phase. In addition, combination of *let-7b* mimic with paclitaxel or gemcitabine notably increased the apoptotic sub-G_1_ population in both cell lines, consistent with increased apoptosis observed in Fig 3C and 3D.

Next, the colony formation assay was carried out to further characterize the effect of *let-7b*/cytotoxin combination on the proliferative capacity of tumor cells. As shown in Fig 4C and 4D, the number and size of colonies formed by A549 and Panc-1 cells were drastically decreased following the treatment of *let-7b*/paclitaxel or *let-7b*/gemcitabine, compared to the individual agents. Taken together, these results support the notion that *let-7b* synergizes with cytotoxins to arrest cell cycle and inhibit the proliferation of *KRAS* mutant tumor cells.

### Combination of *let-7b* with cytotoxins markedly reduces migration and invasion of *KRAS* mutant tumor cells

**Fig 5:**
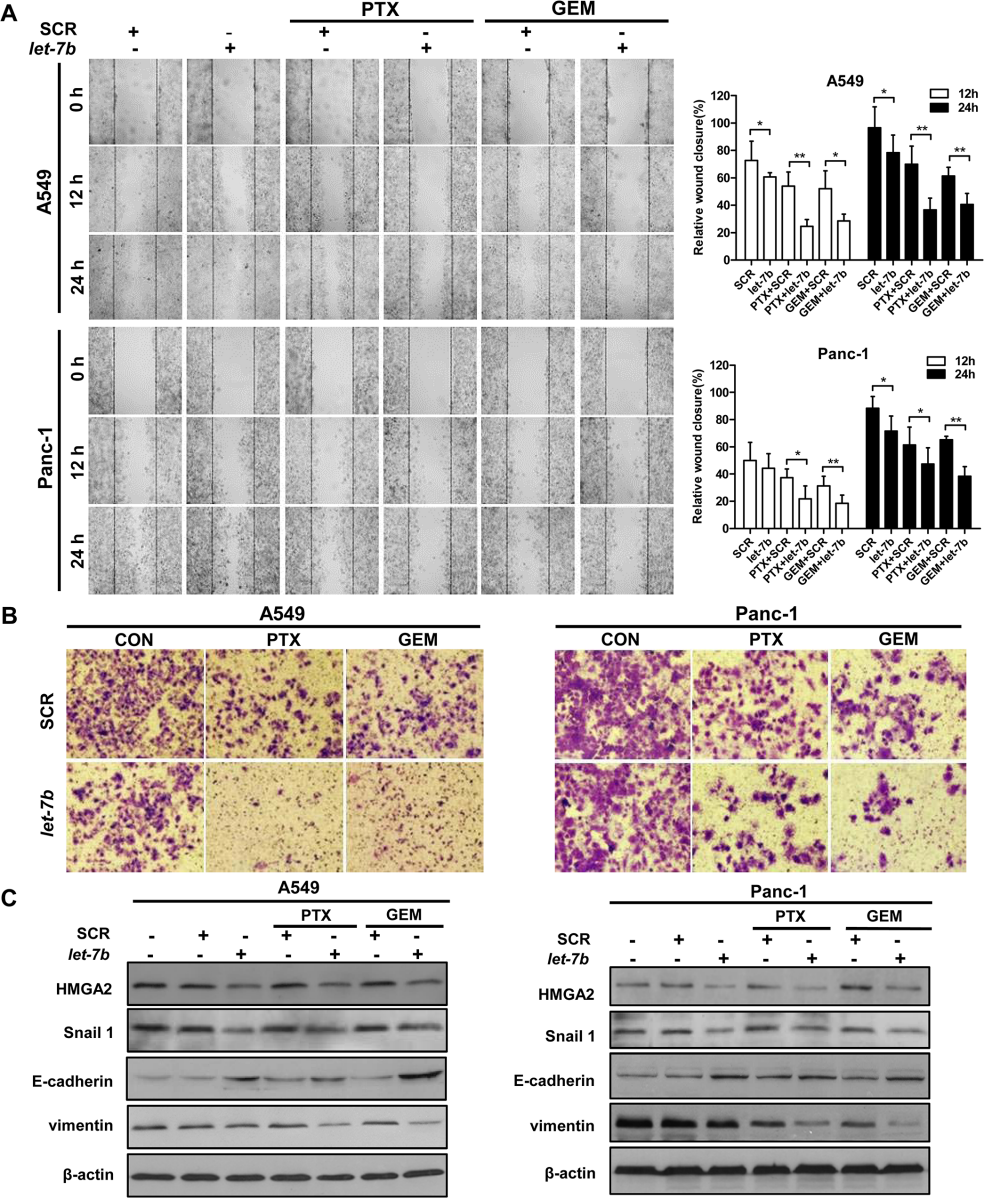
*Let-7b*/cytotoxin combination reduces migration and invasion of *KRAS* mutant tumor cells. A, the effect of *let-7b* mimic, paclitaxel or gemcitabine on cell migration was evaluated in A549 and Panc-1 cells at 0, 12 and 24 hours following the scratch wound (images, left panel). The area of wound healing was quantified by ImageJ (right panel). B, the matrigel invasion assay was conducted to evaluate the effect of *let-7b* mimic, paclitaxel or gemcitabine on the invasiveness of A549 and Panc-1 cells. Images were acquired using an inverted microscope at × 40 magnification. C, Protein levels of HMGA2, Snail 1, E-cadherin and vimentin in A549 and Panc-1 cells were assessed by Western blotting. Each experiment was carried out at least 3 times. Data represent the mean ± SEM. *, *p* < 0.05; **, *p* < 0.01.

Both A549 and Panc-1 cell lines are well characterized for their high motility [24,25], which is reflective of the metastatic nature of NSCLC and PDAC. To investigate the effect of *let-7b/*cytotoxin combination on migration, we performed the scratch wound healing assay. A scratch wound was created at 48 hours post *let-7b*/cytotoxin treatment as indicated by the black lines, whereas the wound closure was measured based on the gap area filled by the migrating tumor cells. As shown in Fig 5A, A549 and Panc-1 cells treated with the scramble control had over 90% wound closure by 24 hours. Treatment of paclitaxel or gemcitabine alone caused 30–40% reduction in the wound closure, whereas over 50–60% decrease in the wound closure was observed in the cells treated with *let-7b*/paclitaxel or *let-7b*/gemcitabine combination (*p* < 0.05). To further evaluate the effect of *let-7b*/cytotoxin treatment on the invasiveness of tumor cells, the matrigel transwell invasion assay was performed. We found that the invasion of A549 cells was ablated when paclitaxel or gemcitabine was combined with *let-7b* mimic (Fig 5B). By contrast, either agent individually only moderately reduced the invading cell population. Similar results were observed in Panc-1 cells.

EMT is an important process during tumor cell migration and invasion, which converts adherent epithelial cells to motile mesenchymal cells [26]. *Let-7* is known to directly target HMGA2, an important transcription factor that regulates EMT [27]. We found that *let-7b* repletion repressed HMGA2 protein in A549 and Panc-1 cells, which was accompanied by an increase in the expression of epithelial marker E-cadherin and the coordinated decrease in the mesenchymal markers Snail 1 and vimentin (Fig 5C). It is worth noting that the combined treatment of *let-7b* mimic with paclitaxel or gemcitabine reduced vimentin level more drastically than either agent individually. Collectively, these results indicate that combination of *let-7b* repletion with paclitaxel or gemcitabine greatly compromises the migratory ability and invasiveness of *KRAS* mutant tumor cells and reverts the EMT phenotype.

### Chemosensitization of *KRAS* mutant cells by *let-7b* is associated with the suppression of TUBB3 and RRM2

**Fig 6:**
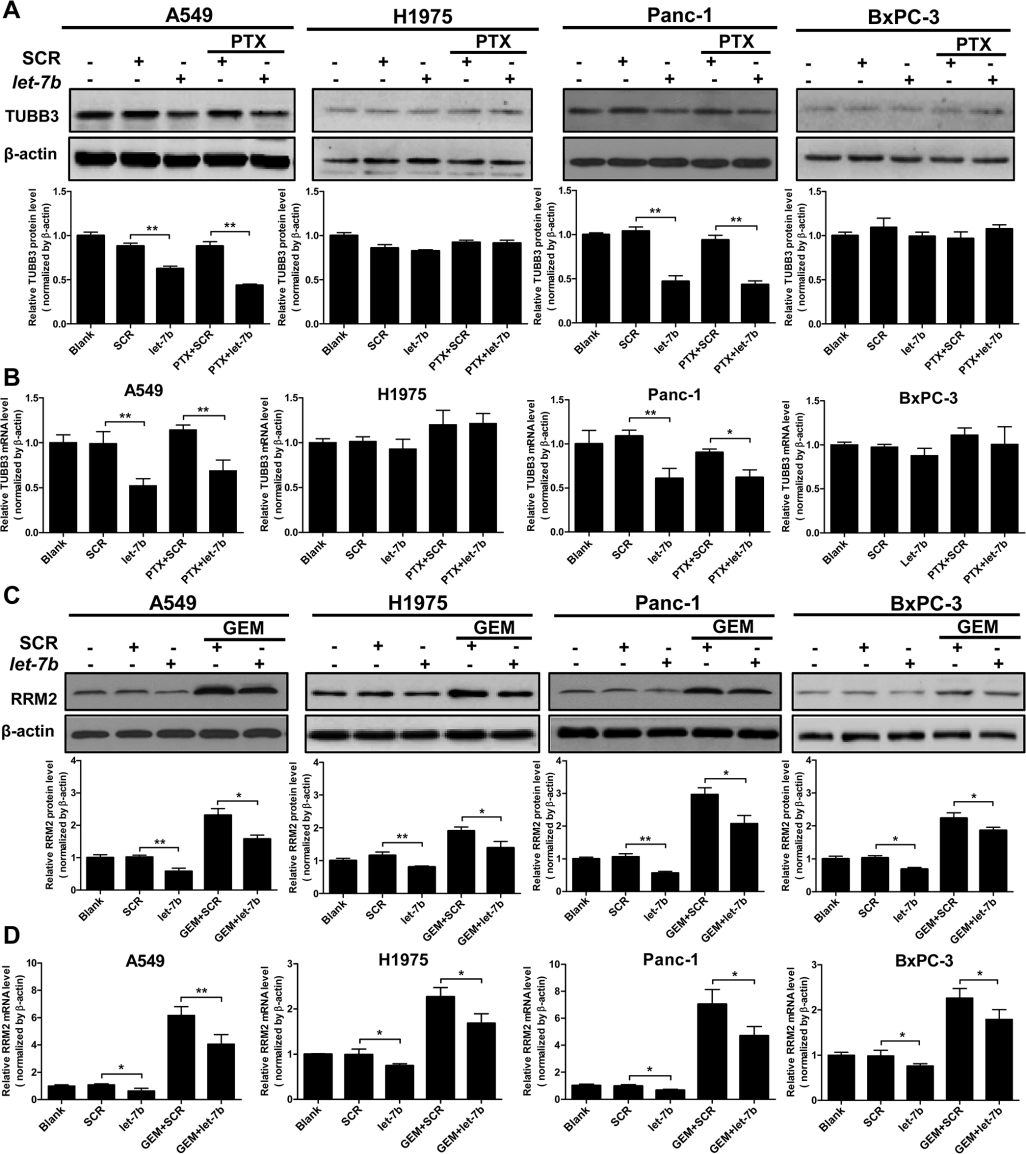
*Let-7b* chemosensitization of *KRAS* mutant cells is associated with the suppression of TUBB3 and RRM2. A, the effect of *let-7b* mimic, paclitaxel or gemcitabine on TUBB3 protein in A549, NIH-H1975, Panc-1 and BxPC-3 cells was analyzed by Western blotting. B, the effect of *let-7b* mimic, paclitaxel or gemcitabine on TUBB3 mRNA in A549, NIH-H1975, Panc-1 and BxPC-3 cells was analyzed by qRT-PCR. C, the effect of *let-7b* mimic, paclitaxel or gemcitabine on RRM2 protein in A549, NIH-H1975, Panc-1 and BxPC-3 cells was analyzed by Western blotting. D, the effect of *let-7b* mimic, paclitaxel and gemcitabine on RRM2 mRNA in A549, NIH-H1975, Panc-1 and BxPC-3 cells was analyzed by qRT-PCR. Each experiment was carried out at least 3 times. Data represent the mean ± SEM. *, *p* < 0.05; **, *p* < 0.01.

TUBB3 is one of the β-tubulin subtypes that has low abundance in most normal tissues but is highly expressed in several solid tumors including NSCLC and PDAC [28,29]. Overexpression of TUBB3 has been correlated with tumor resistance to taxane chemotherapy [30]. To investigate the role of TUBB3 in mediating the sensitization of paclitaxel by *let-7b* mimic, we evaluated the mRNA and protein levels of TUBB3 in the cells exposed to *let-7b*/paclitaxel treatment. We found that TUBB3 was highly expressed in *KRAS* mutant A549 and Panc-1 cells, but was barely detectable in *KRAS* wild-type NIH-H1975 and BxPC-3 cells (Fig 6A). Transfection of *let-7b* mimic reduced TUBB3 at both the transcriptional and translational levels in A549 and Panc-1 cells (Fig 6A and 6B), which paralleled the decline in KRAS protein caused by *let-7b* restoration (Fig 2E). These findings are consistent with the notion that TUBB3 expression is upregulated by mutant but not wild-type KRAS [29]. Not surprisingly, TUBB3 expression in NIH-H1975 and BxPC-3 cells was not altered by *let-7b* repletion (Fig 6A and 6B). These results suggest that *let-7b* sensitizes *KRAS* mutant tumor cells to the cytotoxicity of paclitaxel in part through the suppression of TUBB3.

Overexpression of RRM2 has been linked to gemcitabine resistance in PDAC. Knockdown of *RRM2* using siRNA has been shown to chemosensitize PDAC cells to gemcitabine [31]. Based on the microarray analysis and the presence of *let-7* complementary sites in the 3’-UTR of the gene, *RRM2* is considered to be a putative target of *let-7* [12]. To study the involvement of RRM2 in the sensitization of gemcitabine by *let-7b* restoration, we analyzed RRM2 expression in response to *let-7b*/gemcitabine treatment. Curiously, we found that RRM2 protein level was elevated (~ 2.5-fold) in all gemcitabine-treated cells (Fig 6C). As shown in Fig 6D, the transcription of RRM2 was markedly induced by gemcitabine treatment, and the induction was more robust in the cells harboring mutant *KRAS* (~ 6-fold) than those with wild-type *KRAS* (~ 2.5-fold). Transfection of *let-7b* mimic clearly attenuated gemcitabine-induced RRM2 expression, even though RRM2 protein and mRNA levels were still above the basal levels found in the untreated cells (Fig 6C and 6D). Given the critical role of RRM2 in mitigating gemcitabine cytotoxicity and the robust induction of RRM2 expression upon gemcitabine treatment, these results suggest that the downregulation of RRM2 by *let-7b* may participate in the sensitization of gemcitabine in *KRAS* mutant cells.

## Discussion

Restoration of *let-7* family members has been previously shown to reduce chemoresistance in tumor cells. For instance, resistance to cisplatin in glioblastoma cells has been associated with low *let-7b* level. Reconstitution of *let-7b* re-sensitized glioblastoma cells to cisplatin by abrogating cyclin D1 [32]. In cisplatin-resistant esophageal squamous cells, transfection of *let-7c* restored the sensitivity to cisplatin by inactivating IL-6/STAT3 pathway [33]. In docetaxel-resistant NSCLC cells, the forced expression of *let-7c* increased the *in vitro* and *in vivo* sensitivity to docetaxel via targeting BCL-xL, which was accompanied by partial reversal of the EMT phenotype [34]. In a recent study by Boyerinas et al., the authors showed that *let-7g* replacement re-sensitized MDR1-overexpressing ovarian cancer cells to the cytotoxicity of paclitaxel via IMP-1-mediated reduction of MDR1 [35]. These studies provide strong evidence that *let-7* repletion can re-sensitize drug-induced chemoresistance, although the sensitization by *let-7* appears to be highly dependent on the cellular context in tumor cells.

In the current study, we uncovered a new role of *let-7b* as a chemosensitizer in drug-naive *KRAS* mutant tumor cells. Given that *let-7* binds to 3’-UTR of KRAS mRNA independent of its mutational status in the coding region, it is intriguing that transfection of *let-7b* mimic only diminished the expression of mutant but not wild-type KRAS mRNA (Fig 2F). While the molecular mechanism accounting for this selectivity remains to be fully elucidated, it is plausible that the downregulation of KRAS expression by *let-7b* is dependent on the stoichiometry between KRAS mRNA and *let-7b* in tumor cells. Compared to *KRAS* wild-type cells, *KRAS* mutant tumor cells of the same tissue origin were shown to express notably higher KRAS mRNA and lower *let-7b* levels (Fig 2A and 2B). Suppression of KRAS is thus likely rate-limited by low *let-7b* level in *KRAS* mutant cells, which becomes pronouncedly accelerated upon *let-7b* repletion and results in decreased KRAS mRNA and protein levels. In contrast, targeting of KRAS mRNA by *let-7b* may already operate at full capacity in *KRAS* wild-type cells and is not subjected to further enhancement in the presence of ectopic *let-7b* level. Nevertheless, the downregulation of mutant KRAS by *let-7b* alone was insufficient to block the proliferation of *KRAS* mutant cells nor did it induce appreciable apoptosis, consistent with the findings that knockdown of mutant *KRAS* using siRNAs had limited antitumor effect *in vitro* and *in vivo* [19,36]. This is because even though depletion of mutant *KRAS* inhibits MEK/ERK signaling, other pathways such as PI3K/AKT signaling can remain activated through *RAS*-independent mechanisms. In fact, MEK inhibition alone by small-molecule inhibitors was ineffective in suppressing *KRAS* mutant tumors. The combination of PI3K and MEK inhibitors on the other hand drastically improved the tumor response in preclinical models [2,3]. The MEK/ERK and PI3K/AKT signaling each promotes cell growth and survival via their own distinct downstream effectors, while they both converge on the BH3 family of proteins that regulate apoptosis [37]. Combined MEK/ERK and PI3K/AKT inhibition is required to effectively trigger apoptosis in *KRAS* mutant tumor cells [2]. We found that combination of *let-7b* repletion with paclitaxel or gemcitabine diminished both MEK/ERK and PI3K/AKT signaling in *KRAS* mutant tumor cells, leading to substantial increase in apoptosis. Concomitant blockage of these two key pathways is thus largely accountable for enhanced chemosensitivity observed in *let-7b*-transfected *KRAS* mutant tumor cells.

Overexpression of HMGA2 is closely correlated with the malignant phenotype and poor prognosis of NSCLC and PDAC [38,39]. HMGA2 has been shown to drive EMT and metastasis of epithelial tumors *in vivo* [40]. The EMT phenotype of tumor cells is associated with drug resistance to conventional chemotherapy including gemcitabine and paclitaxel, as well as to the molecularly targeted therapies [41–43]. As a transcription factor, HMGA2 cooperates with transforming growth factor β in inducing Snail 1 expression, a zinc-finger transcription factor crucially involved in EMT and tumor progression [44]. By directly binding to the E-boxes of the human *E-cadherin* promoter, Snail 1 represses the transcription of E-cadherin, a key adhesion protein that is typically lost in epithelial tumor cells during EMT [45]. While HMGA2 is a direct target of *let-7*, ectopic expression of HMGA2 partially rescued the growth inhibition by *let-7* [27]. Interestingly, HMGA2 mRNA also functions as a competing endogenous RNA that impedes *let-7* regulation of other oncogene targets [46]. In addition, HMGA2 is involved in maintaining cancer stem cells in the undifferentiated state, which are resistant to most of the cytotoxic drugs [47]. Our results confirm that *let-7b* repletion downregulates HMGA2 and its major downstream effector Snail 1, resulting in the concomitant increase in E-cadherin and decrease in vimentin expression. We show that the combined treatment of *let-7b* mimic and paclitaxel or gemcitabine markedly suppressed the migration and invasion of *KRAS* mutant cells. The reduced HMGA2 expression and the reversal of the EMT phenotype observed in this study likely contribute to the chemosensitization of tumor cells.

Paclitaxel and gemcitabine inhibit cell proliferation via distinct mechanisms. The finding that *let-7b* repletion sensitized *KRAS* mutant tumor cells to both drugs suggests that in addition to the shared molecular targets, *let-7* possibly also modulates other signaling molecules that synergize specifically with each drug. In NSCLC patients, high TUBB3 expression is associated with poor prognosis, which is regulated by mutant KRAS signaling [28]. Knockdown of *TUBB3* using siRNA has been shown to significantly increase the cytotoxicity of paclitaxel and induce apoptosis in *KRAS* mutant tumor cells [48]. We observed a clear decrease in TUBB3 expression at both the transcriptional and translational levels in *let-7b*-transfected *KRAS* mutant tumor cells, paralleling with the decline of mutant KRAS in these cells. It is plausible that TUBB3 downregulation by *let-7b* may play a causative role in sensitizing *KRAS* mutant cells to paclitaxel. Notably, *TUBB3* is a direct target of *miRNA-200c*, which is an important tumor suppressor that induces epithelial differentiation and reverts EMT in tumor cells [49]. Restoration of *miRNA-200c* markedly sensitizes tumor cells to the microtubule-targeting drugs including paclitaxel.

Ribonucleotide reductase is one of the key determinants of gemcitabine resistance in human cancers. Activity of this enzyme, which catalyzes the reduction of ribonucleoside diphosphates to the corresponding 2’-deoxyribonucleotides (dNTP), the building blocks for DNA synthesis, is modulated by its M2 subunit RRM2 [50]. Overexpression of RRM2 expands the dNTP pool and confers gemcitabine resistance to tumor cells [51]. In gemcitabine-resistant tumor cells, RRM2 mRNA is found to be elevated nearly 10-fold relative to that of the parental cells [52]. The RRM2 mRNA level is inversely correlated with the response rate in cancer patients treated with gemcitabine [53]. In the current study, we found that RRM2 expression was rapidly induced by gemcitabine, implying that the upregulation of RRM2 expression in tumor cells is an early event in acquiring resistance to gemcitabine. Attenuation of RRM2 expression by *let-7b* repletion is potentially a promising approach to chemosensitize *KRAS* mutant tumor cells to gemcitabine.

In summary, we have shown that *let-7b* selectively sensitized *KRAS* mutant tumor cells to the cytotoxicity of paclitaxel and gemcitabine. This was accompanied by diminished MEK/ERK and PI3K/AKT signaling, the reversal of the EMT phenotype, and the downregulation of HMGA2, TUBB3 and RRM2. These results have important implications for the treatment of *KRAS* mutant tumors. Currently, the response rate of paclitaxel in NSCLC patients is about 26%, while gemcitabine is effective in about 10% of the PDAC patients [54,55]. Increasing the sensitivity of these tumors to chemotherapy should significantly improve the therapeutic outcome and counteract drug resistance. Therapeutic exploitation of *let-7* as a general chemosensitizer for *KRAS* mutant tumors warrants further investigation.

## Supporting Information

S1 Fig *Let-7b* repletion chemosensitizes *KRAS* mutant breast cancer cells. (TIF)

S2 Fig *Let-7b* /cytotoxin combination fails to block MEK/ERK or AKT signaling in *KRAS* wild-type cells. (TIF)

S1 Table Sequences of the qRT-PCR primers. (DOCX)

S2 Table Effect of *let-7b* repletion on the cytotoxicity of paclitaxel and gemcitabine in *KRAS* mutant (MT) and wide-type (WT) cell lines. (DOCX)
