# Supplementary figures and images for: Let-7 Sensitizes KRAS Mutant Tumor Cells to Chemotherapy
Source: PLoS One. 2015 May 6;10(5):e0126653. doi: 10.1371/journal.pone.0126653 (PMC4422443; doi:10.1371/journal.pone.0126653)

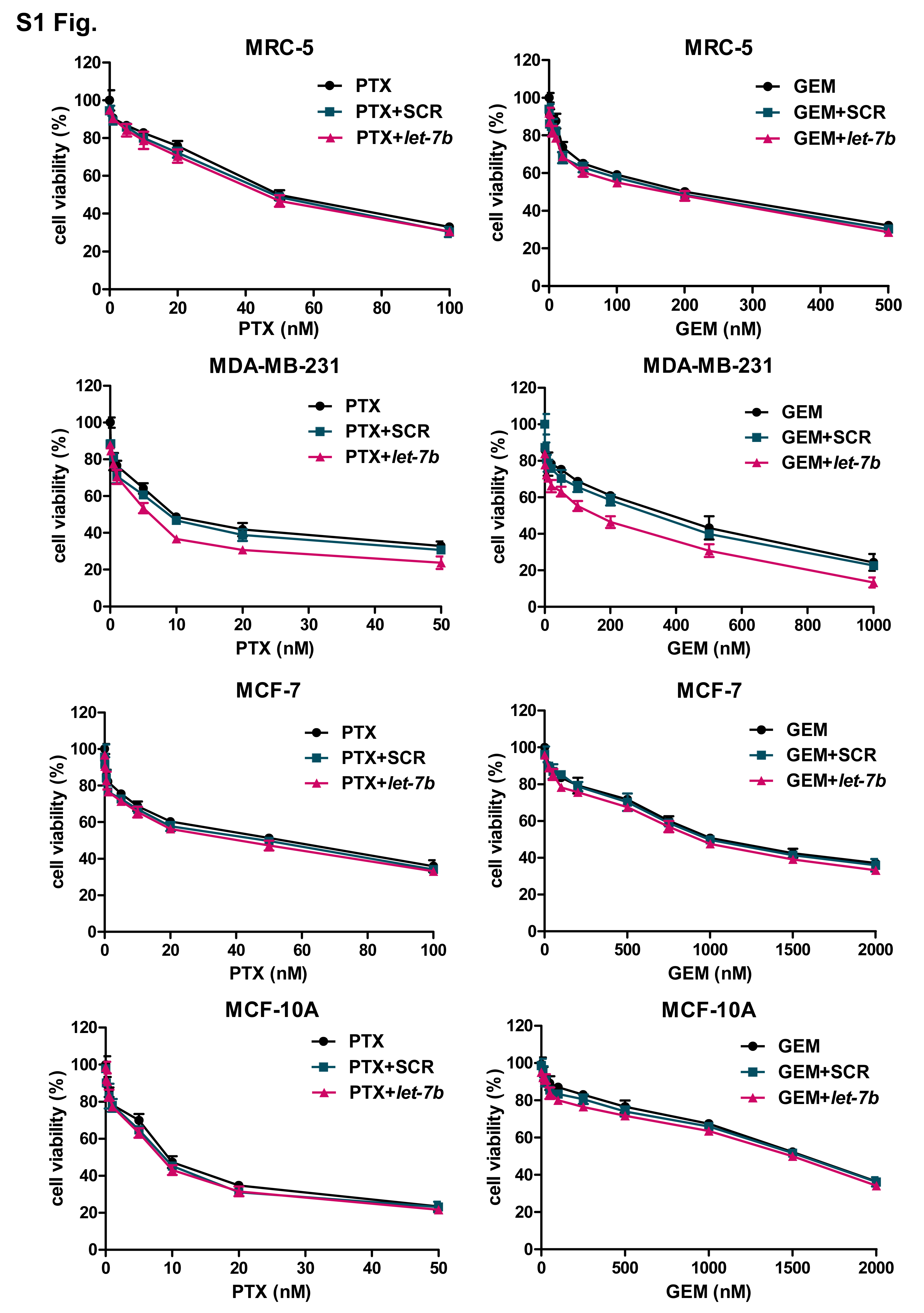

Supplement: S1 Fig — (TIF) [file pone.0126653.s001.tif]

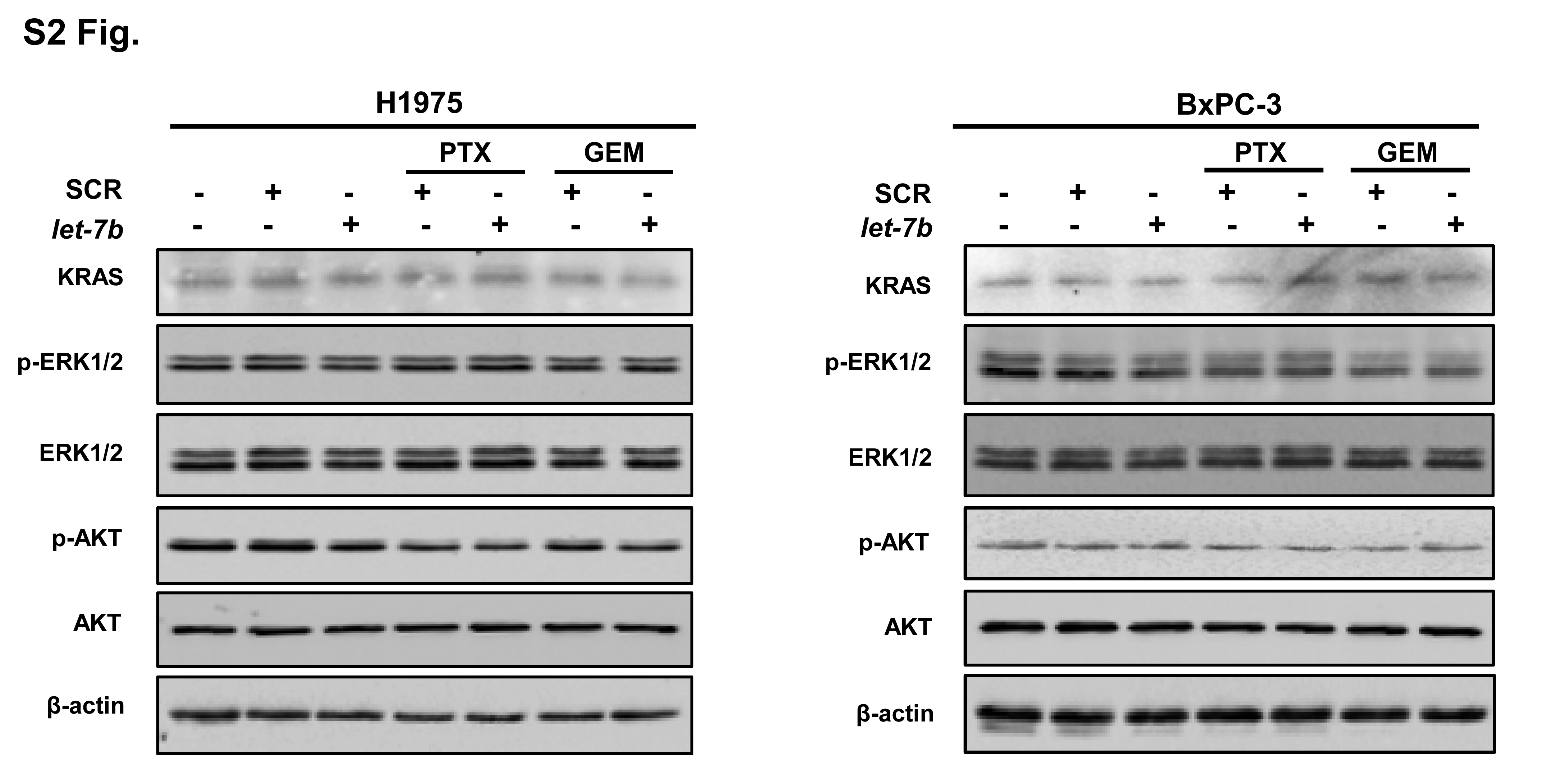

Supplement: S2 Fig — (TIF) [file pone.0126653.s002.tif]
