# Supplementary material for: Let-7 Sensitizes KRAS Mutant Tumor Cells to Chemotherapy
Source: PLoS One. 2015 May 6;10(5):e0126653. doi: 10.1371/journal.pone.0126653 (PMC4422443; doi:10.1371/journal.pone.0126653)
Supplement: S1 Table — (DOCX) [file pone.0126653.s003.docx]

**S1 Table. Sequences of the qRT-PCR primers.**

| **Gene** | **Forward primer sequence** | **Reverse primer sequence** |
| --- | --- | --- |
| *KRAS* | 5'-GACTGAATATAAACTTGTGG-3' | 5'-CTGTTTTGTGTCTACTGTTC-3' |
| *TUBB3* | 5’-CGAAGCCAGCAGTGTCTAAA-3′ | 5′-GGAGGACGAGGCCATAAATA-3′ |
| *RRM2* | 5'-CCCGCTGTTTCTATGGCTTC-3' | 5'-CCCAGTCTGCCTTCTTCTTG-3' |
| *β-actin* | 5'-AGAAAATCTGGCACCACACC-3' | 5'-CTCCTTAATGTCACGCACG-3 |
