# Supplementary material for: Let-7 Sensitizes KRAS Mutant Tumor Cells to Chemotherapy
Source: PLoS One. 2015 May 6;10(5):e0126653. doi: 10.1371/journal.pone.0126653 (PMC4422443; doi:10.1371/journal.pone.0126653)
Supplement: S2 Table — (DOCX) [file pone.0126653.s004.docx]

**S2 Table. Effect of *let-7b* repletion on the cytotoxicity of paclitaxel and gemcitabine in *KRAS* mutant (MT) and wide-type (WT) cell lines.**

| **Cell line** | **IC_50_ of Paclitaxel** | | | **IC_50_ of Gemcitabine** | | |
| --- | --- | --- | --- | --- | --- | --- |
|  | **+ scramble** | ***+ let-7b*** | **CI** | **+ scramble** | **+ *let-7b*** | **CI** |
| **MDA-MB-231 (*KRAS* MT)** | 10 ± 1 nM | 4 ±1 nM | 0.61 | 224 ± 4 nM | 92± 2 nM | 0.44 |
| **MCF-7 (*KRAS* WT)** | 36 ± 1 nM | 33 ± 1 nM | 1.01 | 1071 ± 12 nM | 975 ± 5 nM | 0.99 |
| **MCF-10A (*KRAS* WT)** | 7.8 ± 1 nM | 7.3 ± 1 nM | 1.02 | 1625 ± 14 nM | 1425 ± 11 nM | 0.98 |
| **MRC-5 (*KRAS* WT)** | 46 ± 1 nM | 44 ± 1 nM | 1.01 | 142 ± 3 nM | 133 ± 4 nM | 0.96 |

CI: combination index; CI < 0.9 indicates synergism; CI = 0.9–1.1 indicates additivity, and CI > 1.1 indicates antagonism.
